# Workshop on challenges, insights, and future directions for mouse and humanized models in cancer immunology and immunotherapy: a report from the associated programs of the 2016 annual meeting for the Society for Immunotherapy of cancer

**DOI:** 10.1186/s40425-017-0278-6

**Published:** 2017-09-19

**Authors:** Andrew Zloza, A. Karolina Palucka, Lisa M. Coussens, Philip J. Gotwals, Mark B. Headley, Elizabeth M. Jaffee, Amanda W. Lund, Arlene H. Sharpe, Mario Sznol, Derek A. Wainwright, Kwok-Kin Wong, Marcus W. Bosenberg

**Affiliations:** 1 Rutgers Cancer Institute of New Jersey and Rutgers Robert Wood Johnson Medical School, New Brunswick, NJ 08901 USA; 2 The Jackson Laboratory for Genomic Medicine, Farmington, CT 06032 USA; 3 Knight Cancer Institute at Oregon Health & Science University, Portland, OR 97239 USA; 4 Novartis Institutes for BioMedical Research, Inc., Cambridge, MA 02139 USA; 5 University of California San Francisco, San Francisco, CA 94143 USA; 6 Sidney Kimmel Comprehensive Cancer Center at Johns Hopkins University, Baltimore, MD 21287 USA; 7 Oregon Health & Science University, Portland, OR 97239 USA; 8 Harvard Medical School, Boston, MA 02115 USA; 9 Yale School of Medicine, New Haven, CT 06520 USA; 10 Department of Neurological Surgery, Northwestern University Feinberg School of Medicine, Chicago, IL 60611 USA; 11 Dana-Farber Cancer Institute, Harvard Medical School, Boston, MA 02215 USA; 12 Yale School of Medicine, 15 York Street, St LMP 5031, New Haven, CT 06510 USA

**Keywords:** Mouse models, Immunocompetent, Mouse-in-mouse, Humanized mouse, Tumor microenvironment, Cancer immunotherapy

## Abstract

Understanding how murine models can elucidate the mechanisms underlying antitumor immune responses and advance immune-based drug development is essential to advancing the field of cancer immunotherapy. The Society for Immunotherapy of Cancer (SITC) convened a workshop titled, “Challenges, Insights, and Future Directions for Mouse and Humanized Models in Cancer Immunology and Immunotherapy” as part of the SITC 31st Annual Meeting and Associated Programs on November 10, 2016 in National Harbor, MD. The workshop focused on key issues in optimizing models for cancer immunotherapy research, with discussions on the strengths and weaknesses of current models, approaches to improve the predictive value of mouse models, and advances in cancer modeling that are anticipated in the near future. This full-day program provided an introduction to the most common immunocompetent and humanized models used in cancer immunology and immunotherapy research, and addressed the use of models to evaluate immune-targeting therapies. Here, we summarize the workshop presentations and subsequent panel discussion.

## Introduction

Translating preclinical findings into meaningful clinical outcomes can be a costly and inefficient process, as evidenced by the fact that approximately 85% of oncology drugs to enter clinical testing fail to gain approval by the U.S. Food and Drug Administration (FDA) [1]. There is a pressing need to develop preclinical models that will accurately predict efficacy and toxicity prior to in-human clinical testing. In order to advance understanding of the current status and future directions of mouse and humanized models used in cancer immunology and immunotherapy research, SITC held a workshop as a part of the SITC 31st Annual Meeting and Associated Programs on November 10, 2016. This workshop provided an overview of current models used in the field, with a focus on accurately modeling the tumor microenvironment (TME), as well as the use of murine models to evaluate the efficacy and toxicities of immune-targeting therapies. The program concluded with an open panel discussion driven by questions from the audience.

## Meeting report

### Introduction to models of immunotherapy

#### Major questions related to immunotherapies that require models to address

Mario Sznol, MD (Yale School of Medicine) opened the session with a presentation on clinical issues with immune-based approaches that will require preclinical models to address. In his presentation, Dr. Sznol summarized the factors that contribute to development of cancer and can later determine the response to therapy, including host genetics, lifetime environmental exposures, T cell receptor (TCR) repertoire, carcinogenesis, and evolution of the tumor and tumor-host immune relationship.

Inhibition of the PD-1/PD-L1 pathway has shown broad clinical activity across a variety of malignancies. However, only a portion of patients respond to anti-PD-1/L1 therapies, and appropriate animal models are needed to identify additional targets in order to increase response rates. The need to better understand the biology of response and the effect of the TME is evident in the large number of trials recently initiated to test combination approaches in unselected patient populations. Dr. Sznol highlighted areas for future investigation, including the need to identify the antigens recognized by antitumor T cells, understand mechanisms governing T cell infiltration into tumors, define the influence of tumor biology on antitumor immune response, and determine whether other immune cells (e.g., natural killer [NK] cells, NK T cells, B cells, etc.), inhibitory pathways, or antibodies are capable of eliciting an antitumor response. Dr. Sznol concluded by presenting an ideal scenario in which tumor types would be matched to a specific animal model in order to investigate clinical efficacy and predict the toxicity of novel therapeutic interventions.

#### Overview of mouse-mouse models

Marcus Bosenberg, MD, PhD (Yale School of Medicine) presented an overview of immunocompetent mouse-in-mouse models used in cancer immunotherapy research, including genetically engineered mouse models (GEMMs), chemically induced models, and syngeneic graft models. He highlighted the types of currently available models, their utility, the strengths and weaknesses of each model, and ways to improve upon current systems (Table 1). In doing so, Dr. Bosenberg emphasized that models can be used both to understand the basic biology of the immune system and to test novel immunotherapies in predictive models. Both aspects will be important to drive the field forward; however, developing reliable models to predict clinical outcome in humans may be more difficult.

**Table 1:** Mouse-in-mouse models

Model	Examples	Characteristics	Possible Improvements
Genetically engineered (GEMMs)	• Transgenic • Knock-in/out	• Long latency • Incomplete penetrance • Few somatic mutations • Physiological mitotic rate and tumor microenvironment • Low rate of metastasis • Difficult to induce effective immune responses • High bar for therapies being tested and potentially good model to mimic immunologically incompetent tumors	• Increasing antigenicity ○ Mutator alleles ○ Chemical carcinogenesis ○ Model antigens • Enhanced immune backgrounds
Chemically induced	• 3′methylcholanthrene (MCA)	• Fully penetrant • Variable latency • Unclear histological cancer type • High number of somatic mutations • Can be very immunogenic • Often used as syngeneic grafts	
Syngeneic	• Engraftment of mouse cancer cell lines ○ B16, MC38, CT26, RMA, YUMM, etc.	• Easy, inexpensive, and fast to use • Typically subcutaneous injection of cells • Tumor can grow very quickly • Variable immunogenicity • Variable response to immunotherapy • Hard to compare across models • Drive genes are frequently unknown • Contribution of endogenous retrovirus is not known • Mutation burden is frequently high	• Use multiple lines driven by human-relevant genetic changes • Series of similar lines with variable mutational burden • Ability to evaluate antigen-specific responses • Advanced imaging available to follow immune responses sequentially • Evaluate anti-tumor response at metastatic sites • Make lines from inbred cells using CRISPR

Dr. Bosenberg also highlighted work from his group on the development of a variety of Yale University Mouse Melanoma (YUMM) syngeneic cell lines that exhibit high somatic mutational burden [2], some of which will be available from American Type Culture Collection (ATCC) within the next few months. One of the lines, YUMMER1.7 (YUMM Exposed to Radiation), has been shown to regress after a brief period of growth in a wild type (WT) C57BL/6 background. This regression can be overcome by injecting high numbers of YUMMER1.7 cells, although previously injected mice develop CD4 + − and CD8 + −dependent immunity against higher doses of tumor challenge [3]. Moreover, tumors generated from the YUMMER1.7 line are titratable and respond to immune checkpoint inhibition. Dr. Bosenberg concluded by reviewing pathological features of the melanoma tumors in these models, including early myeloid infiltration, T cell infiltration at day 7, immune mediated killing at day 8, and tumor regression versus escape by days 15–18.

#### Overview of humanized mice models

Karolina Palucka, MD, PhD (The Jackson Laboratory for Genomic Medicine) began her presentation by providing an overview of the approaches used to generate humanized mice, including adoptive transfer of human immune cells, transplantation of human hematopoietic cells with or without accessory tissues in pre-conditioned immunodeficient hosts, genetic editing of immunodeficient hosts, and genetic editing of immunocompetent mice. Dr. Palucka summarized her group’s work on first generation Onco-Humice, in which human T cells were transplanted into NOD/SCID β2-microglobulin-deficient mice. In this model, breast cancer cells grew rapidly despite the presence of tumor infiltrating lymphocytes (TIL). These experiments led to a model describing the tumor-promoting inflammation observed in breast cancer in which Th2 polarization contributes to the inhibition of an antitumor CD8+ T cell response. Dr. Palucka highlighted complications of this model including the eventual development of graft-versus-host disease (GVHD).

Dr. Palucka presented examples of progress in the field utilizing humanized mice with host modifications, including MISTRG mice [4], MISTRG6 [5], NSG with mutant KIT [6], BAFF for antibody immunity [7], NSG-SGM3 with CSF1-tg for macrophages and IL2-tg for NK cells [8], NSG-FcRg knock-out for intravenous IgG therapy [9], and next generation humanized mice from the Jackson Laboratory [10]. She concluded by outlining current challenges, including considerations for modeling the mouse and thymic environment as well as human T cell maturation and selection. Finally, Dr. Palucka identified practical considerations for making autologous humanized mice, sourcing hematopoietic progenitor cells (e.g., bone marrow, blood, cord blood, induced pluripotent stem cells), and finally accommodating for variations in diverse host microbiomes.

#### Overview of patient-derived Xenograft models

Andrew Zloza, MD, PhD (Rutgers Cancer Institute of New Jersey) concluded the first session with an overview of patient-derived xenograft (PDX) models, which are subsets of humanized mice with patient engraftments that have been used in models of infectious disease, transplant, GVHD models, and cancer. The PDX models used for cancer research are created by transferring dissociated single cells from patient biopsies into immunodeficient mice. Over time, these tumors grow into patient-derived tumors. The advantage of the PDX model system over cell line-derived tumor models is the ability to model diverse tumor types directly from patients and potential retention of non-tumor cells from the human TME [11]. Tumors can also be fragmented instead of dissociated, and surgically transplanted into mice, resulting in rapid tumor growth (vessels will start to infiltrate within 48–72 h). Using this method, real-time testing of therapeutic interventions could be used to inform clinical decisions, although there are advantages and disadvantages when using both fragmentation and dissociation methods to generate PDX models (Table 2).

**Table 2:** Methods used to generate PDX models

Dissociation method	
*Advantages*	*Disadvantages*
• Unbiased representation/sampling of whole tumor (unlike sectioning) • Ability to challenge a large number of mice with primary tumor cells (especially when injected within a supporting/collagen matrix)	• Dissociation capabilities and forces may bias the number and type of cells • The formation of mouse-human hybrid tumor (increases with number of passages) • Tumor microenvironment (TME) is not preserved
Fragmentation method:	
*Advantages*	*Disadvantages*
• TME is maintained (hypoxia, acidity, cell:cell interactions, tissue architecture) • May better reconstitute the immune component to some degree	• Not representative of the entire tumor (spatially segregated subclones and immune cells) • Cannot study early tumor formation and responses

Among the benefits of utilizing PDX models is the ability to study metastasis [12, 13]. In addition, tumors engrafted in the original PDX models can be expanded and passaged into subsequent generations of mice. However, the resulting tumors lose some aspects of the original patient tumor characteristics with each generation [12, 13]. PDX models have also been shown to model patient’s disease course with respect to local and distant metastases as well as overall patient outcomes, illustrating the prognostic value of these models [12, 14]. Of note, there are a variety of organizations that are offering PDX models commercially [15]. Concluding with the future of PDX models, Dr. Zloza highlighted the potential for creating double-humanized mice by engrafting both the patient’s tumor and peripheral blood immune cells. In studies using this combination approach, these models lead to good immune reconstitution and maintain proportions of immune cell populations that reflect that of the patients from which the models are derived. Thus, this technique offers an exciting avenue to directly model the human immune system and the TME.

### Session II: Modeling the tumor microenvironment

#### Evaluation of the tumor microenvironment

The second session of the workshop opened with a presentation by Mark B. Headley, PhD (University of California, San Francisco) that focused on modeling the TME. Dr. Headley began by describing the TME as a complex network of cells (tumor cells, immune cells, fibroblasts, endothelium, etc.) that cross-communicate and modulate the antitumor immune response. Notably, the TME differs by cancer type, patient, lesion, and can even vary within the same lesion. Since immune cells in the TME can support or inhibit tumor growth and survival, an understanding of the TME composition and function of these cells provides important diagnostic and prognostic information. For example, tumor-associated macrophages (TAM) are typically pro-proliferation, pro-angiogenic, pro-metastatic, and immunosuppressive. In contrast, NK cells, conventional CD103+ DC, and effector CD8+ T cells, which also populate the TME, act in an antitumor capacity to protect the host from cancer. Neutrophils can be viewed as having both pro- and anti-tumor functions.

Dr. Headley then presented an overview focusing on the mechanisms that balance the pro- and antitumoral functions of myeloid cell populations [16]. Investigations of primary murine and human tumors revealed a combination of macrophage and DC populations within the TME that arise from distinct cell lineages [17]. These results were used to identify a high-DC gene signature that correlated with better patient outcomes [17]. Intravital imaging illustrated conventional DC-CD8+ T cell interactions in the metastatic and primary tumor draining lymph nodes (LN), and elimination of conventional DC in murine models resulted in increased tumor growth, metastasis, and reduced survival. In both primary and metastatic tumors, conventional DC (likely CD103+) set an equilibrium with macrophages, restricting overall tumor growth and metastasis through activation of CD8+ T cells [18]. Dr. Headley concluded by emphasizing that the analysis of cell populations within the TME can yield critical knowledge of the functions of these distinct cell populations and provide prognostic insight into human disease.

#### Factors affecting tumor – Microenvironment interactions

Historically, mesothelioma is chemotherapy resistant and recent therapeutic advancements have demonstrated only modest improvements in OS compared with previous therapies [19]. Highlighting work from her laboratory on the biology of the TME in the setting of mesothelioma, Lisa M. Coussens, PhD (Oregon Health and Sciences University) described the complexity of the TME, which is typically skewed to a Th2-prosurvial, proinflammatory, pro-angiogenic, profibrotic, immunosuppressive microenvironment that can impede drug delivery and limit response to therapy. Investigations into the cellular composition of human mesothelioma have shown that macrophages are the major immune cell infiltrate present, regardless of the type of chemotherapy or type of mesothelioma [20]. Utilizing multiplex immunohistochemistry, it was found that chemotherapy induces infiltration of CD206+ macrophages that are associated with a Th2/M2 phenotype.

Dr. Coussens’ group used syngeneic mouse models of mesothelioma to determine whether macrophages are a valid therapeutic target in this setting. In light of the fact that the colony-stimulating factor receptor axis (CSF1/CSF1R) is predominantly expressed by macrophages and is required for macrophage maturation [21], and that CSFR1 blockade depleted 50% of macrophages in mice with late-stage disease, the group started by inhibiting the CSF1/CSF1R axis. As monotherapy, reductions in macrophages did not decrease tumor burden or increase survival in the mice. Similarly, although the combination of chemotherapy and CSF1R blockade improved cellular apoptosis, led to an influx of CD8+ T cells, and a 50% reduction in primary tumor burden, these effects did not result in increased survival. Instead, lung metastases were resistant to therapy and although the combination successfully depleted macrophages that were recruited to the lungs, there was no recruitment of CD8+ T cells to the metastases. The addition of a PD-L1 inhibitor to the combination controlled the lung metastases and significantly improved survival compared with combination therapy alone. Dr. Coussens concluded by emphasizing that appropriate modeling is essential to the development of rational combination approaches.

#### Vascular regulation of the tumor microenvironment and immune responses

Amanda Lund, PhD (Oregon Health & Science University) presented work on the role of vascular regulation at the interface of a developing malignancy and the systemic immune response. The vasculature coordinates the trafficking of leukocytes as they become activated and re-enter the site of inflammation to mediate effector functions. However, tumor-associated vasculature is hyperplastic and dysfunctional: it maintains the fluid dynamics of tissue, which can regulate hypoxia, impact drug delivery, and can act as a route of metastasis. These functions are regulated by members of the vascular endothelial growth factor receptor (VEGFR) family that drive the migration, proliferation, and integrity of endothelial cells. Importantly, the endothelial phenotype in T cell inflamed and non-inflamed tumors has been shown to directly inhibit lymphocytes from infiltrating tumors [22]. Thus, reevaluation of the anatomy or the vasculature may provide insights into the barriers encountered by T cell-mediated antitumor immunity, and inspire novel immunotherapeutic approaches to overcome these.

Murine models have proved useful in clarifying the role of the vasculature during an immune response, and lymphatic vessels in particular were found to be necessary for de novo antitumor immunity in an implantable murine melanoma model [23, 24]. Inhibition of VEGF-C/D and absence of dermal lymphatic vessels impaired inflammatory carcinogenesis [25], whereas VEGF-C overexpression in the TME drove lymphangiogenesis and regional immunosuppression [24]. Thus, while necessary for immunity, lymphatic function may also lead to immune dysfunction and suppression when activated in an aberrant manner. Flow cytometry was used to examine both the blood and lymphatic endothelial cells in order to understand this complex dependency. Using this method, it was found that tumor associated lymphatic vessels respond to the changing immunologic context within tumor microenvironments and express various regulatory and adhesion molecules that may influence CD8+ T cell responses. Interactions between inflamed, cutaneous lymphatic vessels and egressing lymphocytes may represent a novel point of immune control. Targeting these barriers may, in combination with immunotherapy, drive immune cell priming, infiltration, retention, and function.

#### Components of the tumor microenvironment that modulate tumor immune responses

Kwok-Kin Wong, MD, PhD (Dana-Farber Cancer Institute, Harvard Medical School) presented work using conditional mouse lung cancer models using intranasal Cre recombinase adenovirus to modulate tumor-relevant genes at specific times, resulting in lung cancer induction with almost complete penetrance. He explained that low mutational load and the low-throughput nature represent limitations of this approach. In the EGFR/KRAS model, PD-1 blockade decreases factors in the TME that are immunosuppressive for these EGFR-driven tumors [26]. In addition, long-term PD-1 blockade results in increased progression-free survival and OS in this model. Unlike humans, these mice develop resistance to PD-1 blockade, which provides the opportunity to investigate changes in the TME that influence mechanisms of resistance.

Dr. Wong presented several approaches to increase the mutational load in next generation GEMMs, to increase their utility in studying the antitumor immune response. In the first approach, KRAS/p53-, KRAS/p53/LKB1-, and EGFR/p53-deficient transplantable cell lines were exposed to irradiation or a carcinogen, or were combined with DNA damage response (DDR) gene inactivation in vitro. These cells were then transplanted orthotopically to study alterations in the immune response. In another technique, an organotypic culture was developed to test combination therapies in a high-throughput fashion [27]. Lung nodules from GEMMs were extracted and seeded into three-dimensional (3D) microfluidics chambers to grow spheres containing malignant cells as well as immune cell populations [28, 29]. This technique allows for a variety of parameters to be measured. Once established in culture, light microscopy can be used to track growth, cytokine analyses can be performed, and fluorescence or confocal microscopy can be used to view cellular interactions in real-time. Moreover, this technique can be performed for murine-derived as well as patient-derived tumor spheres. These data indicate that organotypic tumor spheroids derived from murine models can be used in a high-throughput manner to study the TME and correlate with treatment outcomes in patients.

### Session III: Modeling evaluation of immune therapies

#### Evaluation of immune checkpoint inhibitors in mice

Arlene H. Sharpe, MD, PhD (Harvard Medical School) presented work evaluating immune checkpoint therapies in mouse models. Dr. Sharpe opened her presentation with an overview of the PD-1 pathway, noting that activation of the PD-1 receptor leads to downstream signaling that results in reduced TCR signaling, cytokine production, and target cell lysis [30]. PD-L1 can be expressed on a wide variety of hematopoietic cells, non-hematopoietic cells, and tumor cells in the TME. The function of PD-L1 on tumor cells is not clear; it may reflect an inflamed tumor environment and/or contribute to immunosuppression [31]. To investigate the function of PD-L1 on MC38 tumors, PD-L1 was deleted on MC38 tumor cells and the growth of PD-L1-expressing and PD-L1-deficient tumors was comparable. However, deletion of PD-L1 in MC38 tumors increased susceptibility to clearance. These results were further validated in a mixed competition assay in which PD-L1-sufficient tumor cells were transplanted alongside PD-L1-deficient tumor cells. In these experiments, the tumor cells lacking PD-L1 were selectively eliminated. Thus, PD-L1 on tumor cells has a dominant role in limiting antitumor immunity to MC38 tumors. However, the role of PD-L1 expression on tumors is tumor-dependent. Analogous studies of PD-L1-deleted Brafv600 PTEN-deficient tumors and B16 tumors revealed that PD-L1 expression on host cells has dominant role in limiting immune responses to these tumors. The dominance of PD-L1 on tumors may be influenced in part by the immunogenicity of the tumor.

#### Re-evaluating the role of IDO1 in brain cancer; humanized Immunocompetent mice take center stage

Derek A. Wainwright, PhD (Robert H. Lurie Comprehensive Cancer Center at Northwestern University Feinberg School of Medicine) opened his presentation with an overview of glioblastoma multiforme (GBM), noting that these central nervous system (CNS) tumors are universally fatal and their diffuse nature, heterogeneity, and resistance to cytotoxic monotherapy all contribute to the challenges associated with treatment. Since T cells can infiltrate the CNS, a phenomenon commonly seen in primary glioblastoma [32], Dr. Wainwright’s laboratory utilizes murine models to approximate this aspect of the disease. The most common model for glioblastoma is the syngeneic GL261 orthotopic mouse glioblastoma model in which GL261 glioblastoma cells are stereotactically implanted intracranially. In this model, there is a progressive increase in Treg from one to three weeks during tumor development [33]. However, when B16-F10 cells were used in this model, there was no increase in Treg, indicating that tumor-intrinsic mechanisms drive this infiltration [34]. This finding underscores the significance of Treg in glioblastoma and is functionally validated by increased survival in mice with intracranial glioblastoma and neutralized for Treg infiltrates [33].

Indoleamine 2,3 dioxygenase 1 (IDO1) is an IFN-inducible enzyme that converts tryptophan to kynurenine and has been shown to suppress effector T cell functions and activate and expand Treg [35–40]. The depletion of tryptophan and/or accumulation of kynurenine leads to functional inactivation of CD8+ T cells and/or induction of Treg [41]. In the GL261 model, a substantial increase in survival is seen when mice are intracranially-engrafted GL261 cells stably knocked down for IDO1 expression. This survival advantage is also observed when GL261 cells are injected into mice with a systemic IDO1 deficiency. However, the survival advantage is abrogated when implanted into T cell-deficient mice, highlighting the dual importance of tumor cell IDO1 inhibition, in addition to the presence of an intact immune system for eliciting effective tumor rejection [42]. In humans, high IDO1 mRNA levels are prognostic for decreased GBM patient survival. Notably, increased levels of CD3ε/CD8α mRNA correlate with higher IDO mRNA, suggesting that the presence of T cells regulates IDO1 expression. In the syngeneic mouse model using GL261 cells, simultaneous treatment with standard of care radiotherapy, as well as PD-1 and IDO-1 blockade, synergistically increased survival, durably. Extrapolating these findings to the clinical arena, Dr. Wainwright proposes a combinatorial therapy consisting of radiotherapy plus checkpoint blockade and IDO-1 inhibition for the treatment of adults diagnosed with incurable GBM.

#### Developing new immunotherapies in preclinical models and humans

Elizabeth M. Jaffee, MD (The Sidney Kimmel Comprehensive Cancer Center at Johns Hopkins University) addressed ways to accelerate the development of immunotherapy for resistant or immunologically inert tumors. There are several challenges in treating malignancies that do not respond to current immune checkpoint therapy. First, methods to induce functional effector T cell recruitment must be developed. Each cancer and cancer subtype may have a unique TME, illustrating the need to understand immunosuppressive mechanisms that have a clinical impact. Another characteristic that can indicate a lack of response to immune checkpoint inhibitor therapy is a paucity of effector T cells. In contrast to melanoma, which shows spontaneous infiltration of CD8+ T cells, pancreatic cancers are infiltrated with suppressive Treg and myeloid-derived suppressor cells (MDSC). Combination approaches to address these challenges will require novel trial designs and clinical development pathways to gain regulatory approval by the FDA.

Dr. Jaffee proposed a two-step process for the effective treatment of currently immunotherapy-unresponsive tumors: reprogramming the TME and optimizing the immunotherapeutic modality to generate a lasting antitumor response. Efforts to reprogram the TME should focus on improving tumor antigen presentation and abrogating local immunosuppression [43]. Using work from her group to illustrate these ideas, Dr. Jaffee described a study using the whole tumor cell vaccine, GVAX, in the neoadjuvant and adjuvant setting. In this study, GVAX was given two weeks prior to surgery. Following surgery, the patients went on to receive adjuvant chemotherapy. Two weeks after a single vaccine treatment, biopsies from 85% of patients had peri- and intratumoral lymphoid aggregates with features reminiscent of tertiary lymphoid structures. Upregulation of PD-1 was noted in the macrophage and dendritic cell populations within the lymphoid aggregates, which led to an ongoing trial of neoadjuvant GVAX with or without PD-1 inhibition. The potential for personalized immune checkpoint inhibitor therapy based on individual patient expression of immune checkpoints was also raised.

#### What information provided by models will inform immune drug development and use?

Philip Gotwals, PhD (Novartis Institutes for BioMedical Research, Inc.) provided an industry perspective on information gained from models that help direct drug development and optimize current therapies. Questions to be addressed through basic and translational research include patient selection based on knowledge of resistance and biomarkers, determining optimal therapeutics for a given cancer type, and defining appropriate dosing, sequencing, and combinations of therapy. According to Dr. Gotwals, all the models discussed in this workshop could answer such questions; the difficulty is that there are too few models specific to cancer immunotherapy, and limited availability compared to the large libraries of patient-derived xenograft (PDX) models developed to test targeted genetic mutations.

Dr. Gotwals went on to present work from a few ongoing Novartis initiatives, including chimeric antigen receptor (CAR)-T cell approaches targeting TIM-3 and exploiting the effects of signaling through the stimulator of interferon genes (STING) pathway. The STING study focused on use of syngeneic models to study the antitumor immune effects of activating dendritic cells using STING agonists. ADU-S100, a potent cyclic dinucleotide STING agonist, has been shown to induce an abscopal effect and establish immunological memory in a dual flank model using B16 melanoma cells [44]. Combination approaches have also been used in this setting to illustrate that the abscopal efficacy of ADU-S100 combined with immune checkpoint inhibition is dependent on CD8+ T cells. Currently in phase I to assess the pharmacodynamic effects of ADU-S100 in injected as well as distal lesions, these clinical trials are designed to inform further testing in syngeneic models.

### Session IV: Panel discussion and future directions

#### Future directions for the development and use of cancer immune models

The panel discussion, moderated by Dr. Bosenberg, included all Workshop presenters and was driven by questions from the audience. Highlights included a discussion on the need for paired pretreatment and biopsies while patients are on treatment and responding in order to get a better understand of the mechanisms underlying response. The preference for multiple biopsies in clinical trials was expressed; however, multiple biopsies can raise ethical concerns in addition to considerations of patient compliance and safety. As an alternative to multiple tumor biopsies, patient-derived peripheral blood mononuclear cells (PBMC) could be used in PDX models generated from patient’s tumors. The use of models to predict the timing and sequencing of combination approaches was also discussed, as limitations initially attributed to models may actually be the result of improper sequencing and/or dosing of therapies. Finally, the panel addressed questions regarding the use of models to develop treatments for immunologically inert tumors in which tumor-specific T cells may be present but non-functional. Models are necessary to determine the underlying mechanisms behind this phenomenon, which will be key to developing therapies to treat these diseases.

## Conclusions

Dr. Bosenberg offered concluding remarks and summarized the main themes from the day. Syngeneic models are cost-effective and easy to use; however, GEMM may better approximate the TME and vascular architecture, but tend to have low neoepitope/mutation burden. Advances in humanized mouse models are rapidly progressing, and with time, will hopefully bridge the gap between mouse-in-mouse models and clinical experience. The unique milieu of the TME can have a significant impact on response to therapy via suppressive mechanisms that are not yet entirely understood. Highlighting the diversity and promise of the types of models presented, Dr. Bosenberg emphasized that reliable pre-clinical models will be essential to understanding mechanisms of response as well as resistance to immunotherapy. Although each model has strengths and weaknesses, advances in modeling the dynamic interaction between the immune system and cancer will be critical to advances in the field, particularly in the development of rational combination approaches.

## Abbreviations

3D: Three-dimensional
ATCC: American Type Culture Collection
CAR: Chimeric antigen receptor
CNS: Central nervous system
CSF: Colony stimulating factor
CTL: Cytotoxic T lymphocyte
DC: Dendritic cell(s)
DDR: DNA damage response
FDA: Food and Drug Administration
GBM: Glioblastoma multiforme
GEMMs: Genetically engineered mouse models
GVHD: Graft-versus-host disease
IDO1: Indoleamine 2,3-dioxygenase 1
LN: Lymph node(s)
MDSC: Myeloid-derived suppressor cell(s)
NK: Natural killer cell(s)
OS: Overall survival
PBMC: Peripheral blood mononuclear cell(s)
PD-1: Programmed cell death 1
PDX: Patient-derived xenograft
SITC: Society for Immunotherapy of Cancer
STING: Stimulator of interferon genes
TAM: Tumor-associated macrophage(s)
TCR: T cell receptor(s)
TIL: Tumor infiltrating lymphocyte(s)
TME: Tumor microenvironment
Treg: Regulatory T cell(s)
VEGFR: Vascular endothelial growth factor receptor
WT: Wild type
YUMM: Yale University mouse melanoma
